# Improvement of walking speed and gait symmetry in older patients after hip arthroplasty: a prospective cohort study

**DOI:** 10.1186/s12891-015-0755-3

**Published:** 2015-10-12

**Authors:** Walter Rapp, Torsten Brauner, Linda Weber, Stefan Grau, Annegret Mündermann, Thomas Horstmann

**Affiliations:** Institute of Sport and Sport Sciences, Albert-Ludwigs Universität Freiburg, Schwarzwaldstr 175, 79117 Freiburg, Germany; Conservative & Rehabilitative Orthopedics, Technische Universität München, Munich, Germany; Department of Food and Nutrition and Sport Science, University of Gothenburg, Gothenburg, Sweden; Department of Orthopaedics, University Hospital Basel, Basel, Switzerland; Medical Park St. Hubertus, Bad Wiessee, Germany

**Keywords:** Gait symmetry, Total hip endoprostheses, Inertial sensor, Gait training

## Abstract

**Background:**

Retraining walking in patients after hip or knee arthroplasty is an important component of rehabilitation especially in older persons whose social interactions are influenced by their level of mobility. The objective of this study was to test the effect of an intensive inpatient rehabilitation program on walking speed and gait symmetry in patients after hip arthroplasty (THA) using inertial sensor technology.

**Methods:**

Twenty-nine patients undergoing a 4-week inpatient rehabilitation program following THA and 30 age-matched healthy subjects participated in this study. Walking speed and gait symmetry parameters were measured using inertial sensor device for standardized walking trials (2*20.3 m in a gym) at their self-selected normal and fast walking speeds on postoperative days 15, 21, and 27 in patients and in a single session in control subjects. Walking speed was measured using timing lights. Gait symmetry was determined using autocorrelation calculation of the cranio-caudal (CC) acceleration signals from an inertial sensor placed at the lower spine.

**Results:**

Walking speed and gait symmetry improved from postoperative days 15–27 (speed, female: 3.2 and 4.5 m/s; male: 4.2 and 5.2 m/s; autocorrelation, female: 0.77 and 0.81; male: 0.70 and 0.79; *P <*0.001 for all). After the 4-week rehabilitation program, walking speed and gait symmetry were still lower than those in control subjects (speed, female 4.5 m/s vs. 5.7 m/s; male: 5.2 m/s vs. 5.3 m/s; autocorrelation, female: 0.81 vs. 0.88; male: 0.79 vs. 0.90; *P <*0.001 for all).

**Conclusions:**

While patients with THA improved their walking capacity during a 4-week inpatient rehabilitation program, subsequent intensive gait training is warranted for achieving normal gait symmetry. Inertial sensor technology may be a useful tool for evaluating the rehabilitation process during the post-inpatient period.

## Background

Changes in ambulatory kinematics and kinetics are commonly observed in patients with asymptomatic, moderate and severe osteoarthritis [1] and include changes in stance phase, walking speed, joint moments and joint angular velocities compared to healthy subjects [1, 2]. In particular, the more severe their disease assessed by clinical scores such as Kellgren-Lawrence grade, the slower patients walk [1, 3]. Moreover, several parameters including loading rate and joint angles have been proposed for gait asymmetry assessment in persons with hip osteoarthritis [4] although these authors have raised concern regarding reliability of these parameters. Retraining walking is a major focus of rehabilitation in patients after hip or knee arthroplasty (THA or TKA) especially in older persons whose social interactions are influenced by their level of mobility. Common therapy programs are aimed at improving muscle strength and neuromuscular activation patterns.

Natural gait is characterized by nearly symmetric movement patterns of the lower extremities: able-bodied persons show minimal laterality with only subtle differences between the dominant and non-dominant leg. However, severe gait asymmetries have been observed in patients with hemiplegia [3], Parkinson’s disease [5–7], leg length discrepancies [8] and in lower extremity amputees [9]. While achieving gait symmetry is an important aspect of rehabilitation after lower limb surgery to avoid long-term unilateral loading, to date the required level of symmetry is unknown and is typically clinically evaluated by a patient’s level of pain or discomfort due to their asymmetric gait. Further, diagnoses based on standard clinical methods are subjective and influenced by the physician’s and patient’s perception [10].

Inertial motion devices represent an alternative to laboratory based instrumented gait analysis for objective gait assessments: inertial motion systems are easy to use and over the last decade have become smaller and less expensive, and hence have been increasingly used for many clinical applications including activity monitoring [11] and gait analysis [12, 13]. The stance and stride phases of human walking and running can be reliably calculated from acceleration signals [13]. In particular, the calculation of autocorrelation of the cranio-caudal acceleration signal is a valid method for quantifying gait symmetry in healthy subjects [12]. To date, only few studies [12, 14, 15] have examined symmetry aspects of gait in patients with hip or knee osteoarthritis using these simple devices and showed that these devices are valid for assessing symmetry parameters in these populations [12, 16]. Moreover, these parameters may differ between men and women as the interplay of gait mechanics, pain, and disability differs between men and women with osteoarthritis [17].

The objective of this study was to evaluate the rehabilitation progress in subjects after THA during a 4-week inpatient rehabilitation period using inertial sensor technology. We hypothesized that walking speed and gait symmetry at three different stages of the rehabilitation period incrementally approach values of a reference group of healthy subjects and that a persons sex may influence changes in these parameters.

## Methods

Twenty-nine patients and 30 age-matched healthy subjects (Table 1) participated in this prospective cohort study after providing written informed consent. This study was approved by the official ethics committee of the Medical University Clinic Tübingen (Germany) and followed the principles of the Declaration of Helsinki.

**Table 1:** Anthropometric data of the participants of this study

	Sex	N	Age [years]		Weight [kg]		Height [m]		BMI [kg/m^2^]	
			Mean	SD	Mean	SD	Mean	SD	Mean	SD
THA	female	14	67.8	6.3	70.6	14.3	1.68	.05	24. 9	4.9
	male	15	63.6	7.9	89.4	15.7	1.78	.09	28.1	4.1
RG	female	14	68.0	6.5	67.1	10.0	1.78	.05	25.4	3.4
	male	16	65.9	9.7	86.9	16.6	1.78	.06	27.9	5.2

*THA* total hip arthroplasty, *RG* reference group, *BMI* body mass index, *SD* standard deviation

The THA group comprised patients who were in an inpatient rehabilitation clinic immediately after THA. All subjects in the THA group (15 men, 14 women) received a total hip endoprothesis because of hip osteoarthritis progression with anterior and medial surgical approaches prior to their clinic stay (12 left hips, 17 right hips). Six patients had already received THA on the opposite hip (3 left hips, 3 right hips) at least 1 year prior and were completely pain and symptom free on the contralateral side. Patients were recruited from gait training courses integrated into the clinic’s rehabilitation program and asked to participate in a gait analysis during their stay in the clinic. The inpatient rehabilitation program comprised 4 weeks of daily training with physiotherapy (5 sessions/week), lymph drainage or massage (3 sessions/week), water exercise (3 sessions/week after wound heeling), activity of daily living training (2 sessions/week) and patient education on osteoarthritis and prosthesis (3 sessions). The inclusion criterion for patients was permission from their physician to walk without walking aids and to fully load their operated leg.

A reference group (RG; 16 male and 14 female subjects) aged 50 years or older with similar anthropometrical data were recruited from training courses at the local university clinic. The courses are especially designed for elderly people aimed at improving their physical fitness. Only volunteers without orthopedic disorders at the lower extremities were included.

All subjects were asked to walk on a 20.3-m level walkway at a self-selected preferred walking speed (*normal*). In a second trial, subjects were asked to walk at a fast self-selected speed (*fast*). The individual walking speed was recorded for a 2-m section at the midsection of the walkway using two pairs of photo cells (Alge Timing, Lustenau, Austria). Subjects walked down the track and returned after a short break of 3–5 s. The verbal instructions for both subject groups were identical. Subjects wore their own walking shoes (high-heeled shoes were not allowed) and were asked to use the same pair of shoes for all testing sessions.

Patients with THA performed three test sessions with a minimum of 6 days between each session. The first test day (TD1) was scheduled as soon as the patients felt able to complete the task and when the physician and physiotherapist gave their permission. The first test day was on average (mean (1SD)) 15 (3.5) days post-operatively. The subsequent test days were 21 (3.6) days (TD2) and 27 (3.6) days (TD3) post-operatively. All patients received standard therapy after surgery, and therapists aim at achieving a subjective symmetric gait pattern. Reference subjects completed only one test session because these subjects did not complete any specific training.

### Equipment

Gait analysis was conducted using an inertial sensor unit recording acceleration and gyroscopic signals (Humotion, Münster, Germany). A three-dimensional accelerometer and three orthogonally aligned gyroscopic sensors were integrated into the inertial sensor unit. The system was mounted inside an aluminum box (75 × 70 × 10 mm^3^) and weighed 30 g. Using an elastic belt, the box was secured to the lower part of the dorsal spine at level L4-L5 by the same examiner in each session. This fixation ensured that the measuring axes of the inertial sensor unit closely matched the cardinal body axes. The three acceleration signals represented the medio-lateral, cranio-caudal and anterior-posterior directions, respectively. These axes were also the rotational axes of the gyroscopes. The cranio-caudal acceleration signal was used to detect heel contact, and the medio-lateral acceleration and the medio-lateral gyroscope signals were used to detect left or right foot contact. Two calibration files were recorded while the subjects were (A) standing still in an upright position and (B) leaning their trunk forward to a maximum hip flexion of about 30°. By flexing the hip, the movement orientation of the sensor can be identified from the acceleration signal. Both positions were necessary for offset correction and coordinate transformation to correct for possible orientation errors of the sensor. The vector pointing from the sensor location in the upright position to its position in the forward lean position defined the forward direction regardless of the position of the sensors, and the other two directions were defined orthogonal to this axis. The sensor coordinate system is transformed to match the vertical axis, and hence the calculations are not affected by small deviations in sensor placement on the body.

All signals were recorded at 100 Hz and stored on a chip within the inertial sensor unit. The maximal acquisition time of the inertial sensor was specified by the manufacturer as 24 h and thus did not limit measuring time. Prior to the first measurement, a data file containing relevant patient data was established on the PC-system and on the inertial sensor. Data acquisition began automatically after disconnecting the inertial sensor from the PC-system. After finishing the walking task, the sensor was reconnected to the PC and the stored signals were automatically transferred to the PC for further analysis. All signals were stored in ASCII-format and then imported into a MATLAB 7.1 routine (MathWorks, Germany) for further analyzing.

### Signal processing

**Fig. 1:**
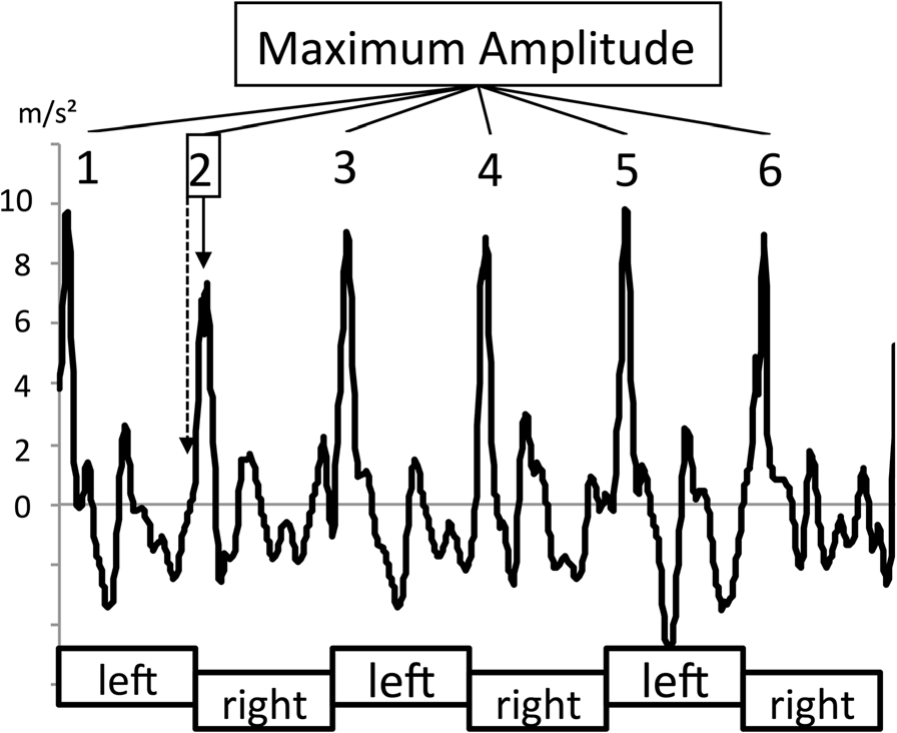
Raw cranio-caudal acceleration signal for 6 steps of one subject. The x-axis was time-normalized to 200 points for each stride (100 points per side). The vertical dotted line represents the trigger set 50 ms before the maximum amplitude for one step

**Fig. 2:**
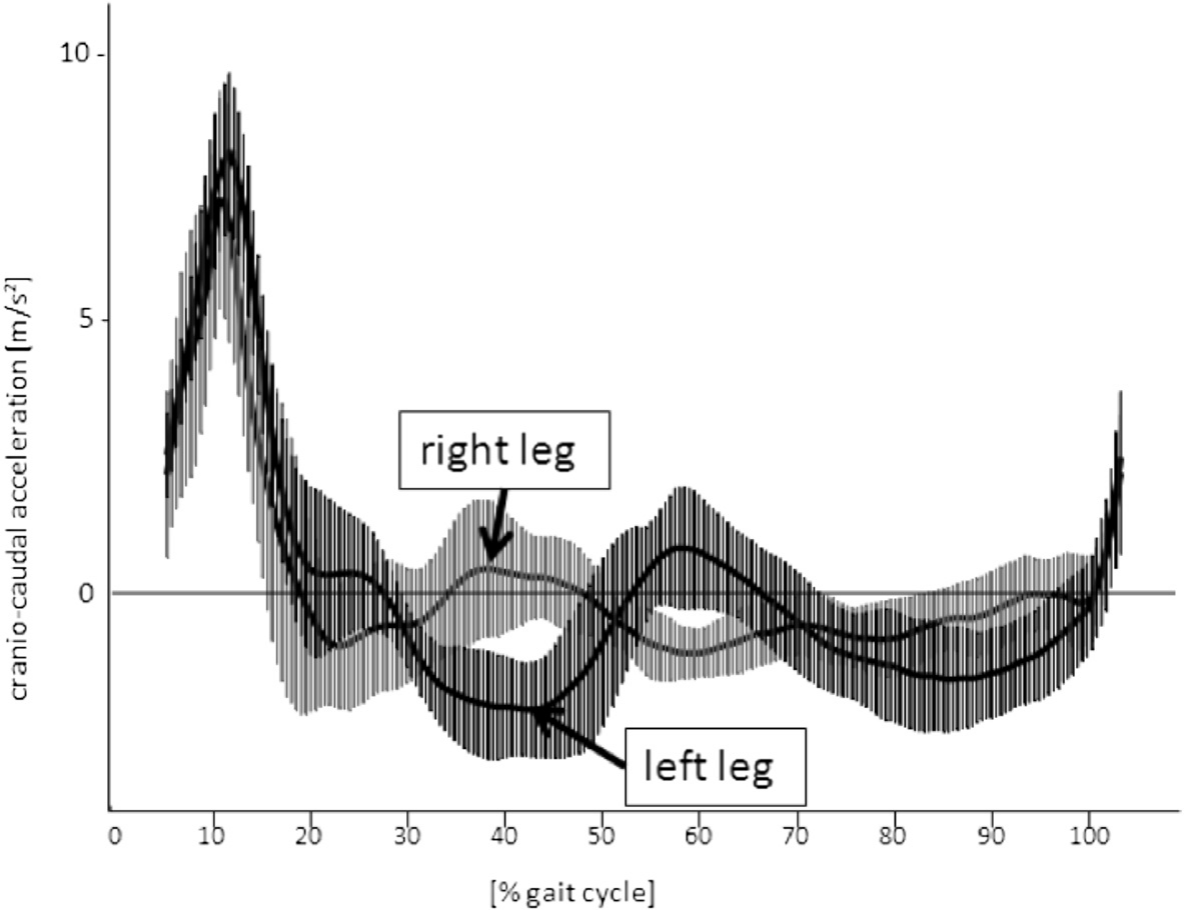
Normalized mean (1SD) cranio-caudal acceleration data for one exemplary patient with THA for the *right* and *left legs* (10 steps per side)

As shown by Auvinet et al. [13] comparing video-based methods with acceleration signals during gait, heel contact is represented by a small peak in the ascending part of the cranio-caudal acceleration signal. While the peak representing foot flat phase is easily detectable, the small peak in the ascending part cannot be easily detected by an automated routine. Our own pilot studies using the inertial sensor unit with synchronized pressure sensitive insoles (Belamed, Germany) confirmed this observation (unpublished data). However, subsequent autocorrelation calculations do not depend on the exact definition of a specific event. To automatically detect heel contact, we selected the maximum peak of the cranio-caudal signal as a trigger with a constant negative delay of 50 ms (Fig. 1), which was used to define individual steps. Because the signal at the beginning and at the end of each measurement was influenced by the subjects accelerating to achieve the desired walking speed, the first and last four steps were eliminated. The acceleration signal for the remaining middle ten strides (ten steps per side) was normalized to 2000 data points so that each step was represented by 100 data points (Fig. 2). All 2000 data points were used for computing autocorrelations.

### Parameter calculation

**Fig. 3:**
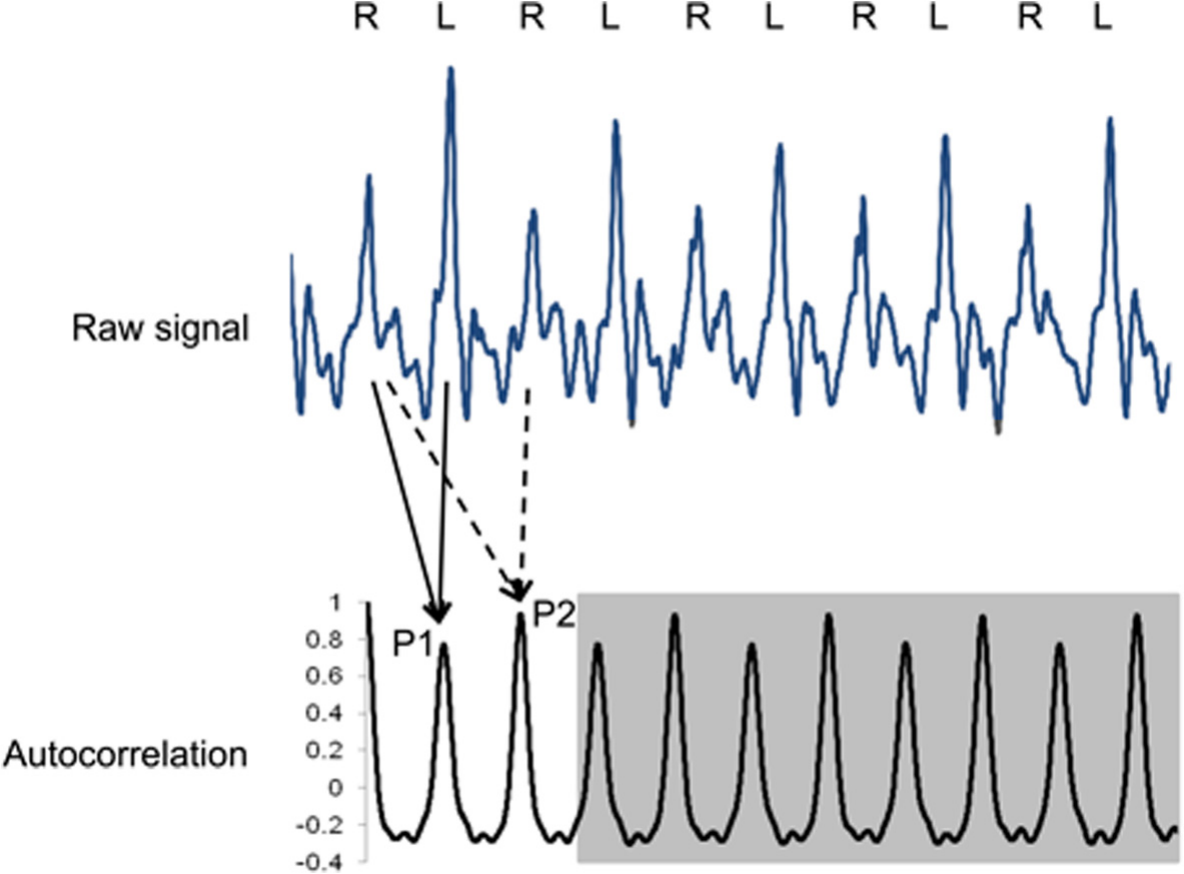
Raw signal (*top*) and computed autocorrelation (*bottom*) for one exemplary trial. P1 represents the regularity between neighboring steps of opposite sides and P2 the regularity between neighboring steps of the same leg

Calculating autocorrelation coefficients has been proposed as valid method for estimating gait symmetry [18–20]. Autocorrelation describes the correlation of a function or signal with itself at an earlier time point. Analyzing a cyclic signal such as a gait pattern produces autocorrelation coefficients with peak values when similar phases overlap. For a time series of the acceleration signal during walking, the first dominant peak (P1) represents a phase shift equal to one step, and the second dominant peak (P2) represent a phase shift equal to one stride. P1 values represent the regularity of neighboring steps and is low if contralateral steps are asymmetric. P2 values represent the regularity of the ipsilateral steps (Fig. 3). P2 values are typically higher than P1 values because ipsilateral steps are more similar than contralateral steps. As recommended by Moe-Nilssen [18], we calculated P1 and P2 from the cranio-caudal signal, and the symmetry index was calculated as the ratio of P1 and P2 (P1/P2).

### Statistic analysis

All statistical analyses were performed using SPSS version 16.0 (IBM Corporation, Somers, NY). A linear mixed model with factors test day (within subjects) and sex and group (between subjects) was used to identify changes in walking speed and gait asymmetry between test days and between groups. Data are presented as group mean values with standard deviations (SD) with test day (TD1, TD2, TD3) as within-subject factors and group (THA, reference) and sex (male, female) as between-subject factors. Independent Student’s t-tests and Least Significant Difference (LSD) tests were used for posthoc analyses. Bonferroni adjustments were applied to posthoc analyses to account for multiple comparisons. The level of significance for all statistical tests was set a priori to *P <*0.05.

## Results

### Walking speed

The linear mixed model revealed significant main effects for test day, sex and group for walking speed (*P* <.001 for all). Walking speed increased from TD1 to TD3 in patients with THA (*P* <0.05; Table 2). While female patients with THA walked slower than age-matched female reference subjects for both speed conditions, male patients with THA walked significantly slower than age-matched male reference subjects only at TD1 and TD2 but not at TD3.

**Table 2:** Mean (1 standard deviation) walking speed and autocorrelation coefficients P1 and P2. The MEAN value represents the average of values at normal and fast walking speeds

			TD1		TD2		TD3		Reference group	
Parameter	Sex	Walking speed	Mean	SD	Mean	SD	Mean	SD	Mean	SD
Walking speed [m/s]	female	normal	2.77	.67	3.75	.54	4.09	.53	5.06	.78
		fast	3.62	.77	4.50	.69	4.83	.70	6.27	.82
	male	normal	3.63	.84	4.20	.47	4.62	.40	4.69	.64
		fast	4.68	.76	5.30	.46	5.75	.52	5.83	.84
Autocorrelation coefficient P1	female	normal	.7738	.0659	.7962	.0883	.8143	.0824	.8828	.0647
		fast	.7604	.0980	.7848	.0988	.8064	.0891	.8771	.0863
		MEAN	.7671	.0820	.7905	.0936	.8104	.0858	.8800	.0755
	male	normal	.6704	.1190	.7350	.1260	.7886	.1246	.9031	.0613
		fast	.7214	.1166	.7597	.1125	.7963	.1037	.8882	.0639
		MEAN	.6959	.1178	.7474	.1193	.7925	.1142	.8957	.0626
Autocorrelation coefficient P2	female	normal	.9007	.0604	.9002	*.0669*	.9120	*.0577*	.9094	*.0680*
		fast	.8983	.0776	.8904	*.1005*	.9003	*.0757*	.9054	*.0888*
		MEAN	.8995	.0690	.8953	*.0837*	.9061	*.0667*	.9074	*.0784*
	male	normal	.8969	.0673	.9174	*.0538*	.9190	*.0564*	.9166	*.0700*
		fast	.9144	.0485	.9198	*.0466*	.9223	*.0572*	.9337	*.0510*
		MEAN	.9056	.0579	.9186	*.0502*	.9206	*.0568*	.9251	*.0605*

*P1* first dominant peak of the acceleration signal, *P2* second dominant peak of the acceleration signal, *SD* standard deviation

In female patients with THA, normal walking speed increased from TD1 to TD2 (*P =* 0.001) and from TD1 to TD3 (*P <*0.001; Table 2). Fast walking speed significantly increased from TD1 to TD2 to TD3 (*P <*0.03 for all). In male THA patients, normal and fast walking speeds significantly increased from TD1 to TD2 to TD3 (*P <*0.03 for all; Table 2).

In general, female patients with THA walked slower than male patients. The largest difference in normal or fast walking speed between sexes was 23 % at TD1. At TD2 and TD3 these differences were between 10 and 15 % (all *P* <.05).

### Gait asymmetry

The linear mixed model revealed significant main effects for test day, sex and group for autocorrelation coefficients for P1 (*P* <.028 for all). Although the autocorrelation coefficients for P1 increased from TD1 to TD3 in patients with THA, the autocorrelation coefficients for P1 on all test days and at both walking speeds were significantly lower in patients with THA than in the reference subjects (*P* ≤0.036 for all). The autocorrelation coefficients for P1 differed between sexes (*P* = 0.028) and test days (*P* = 0.021). The increase in the autocorrelation coefficients for P1 in patients with THA was only significant for male patients at normal and fast walking speed between TD1 and TD3 (*P =* 0.004) and between TD2 and TD3 (*P =* 0.030). There was no interaction between sex and test day (*P* = 458).

The autocorrelation coefficients for P2, representing the correlation between ipsilateral steps, did not differ significantly between test days, sexes, or between patients with THA or reference subjects (*P >*0.050; Table 2).

**Fig. 4:**
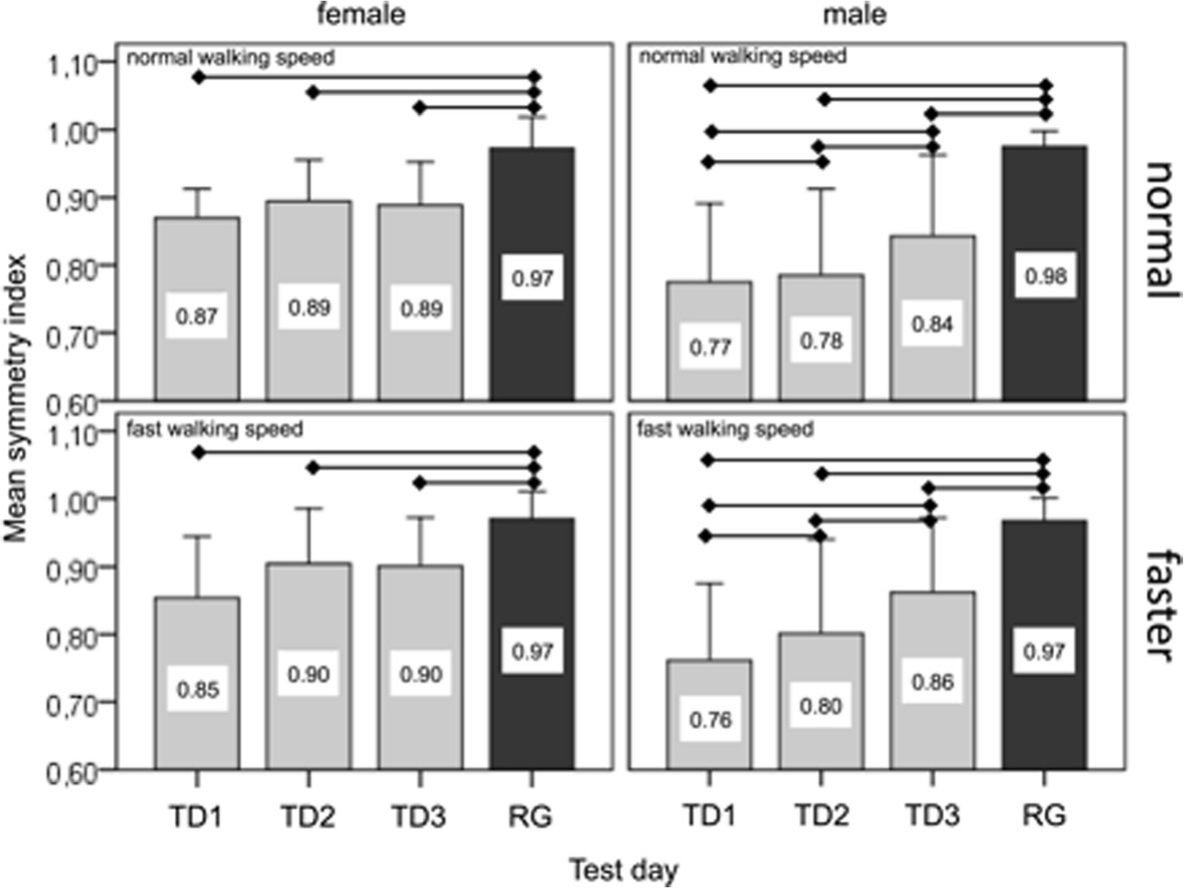
Mean (1SD) symmetry index on three post-THA test days for female and male patients for normal and fast walking speeds. Black bars represent the reference group. TD1—15-days post-THA; TD2—21-days post-THA; TD3—27-days post-THA; THA—total hip arthroplasty. Horizontal bars represent significant differences (*P* <0.05)

The linear mixed model revealed significant main effects for test day, sex and group for symmetry index (*P* <.039 for all). The symmetry indices in patients with THA on all test days at both walking speeds were significantly lower than in the reference group (*P* ≤0.001; Fig. 4). The symmetry index differed between sexes (*P* = 0.003) and test days (*P* = 0.039). In male patients with THA, the symmetry index increased from TD1 to TD2 to TD3 at both walking speeds (TD3 vs. TD1: *P <*0.001; TD3 vs. TD2: *P =* 0.020). The symmetry indices in female patients with THA were higher than those for male patients on all test days at normal walking speed (TD1: *P =* 0.019; TD2: *P =* 0.007; TD3: *P =* 0.214) and at fast walking speed (TD1: *P =* 0.025; TD2: *P =* 0.022; TD3: *P =* 0.268). There was no increase in symmetry index over time in female patients with THA.

## Discussion

The purpose of this study was to evaluate the rehabilitation progress in subjects after THA during an inpatient rehabilitation period using inertial sensor technology. We found that patients with THA had a clear improvement in walking speed after an intensive inpatient rehabilitation period for an average of 27 days. The inertial sensor data showed that asymmetries in a gait cycle decreased over the rehabilitation period. However, especially female patients did not achieve walking speeds of a reference group within the observed rehabilitation period.

Walking speed is an important gait parameter: faster walking speeds reflect greater mobility and capacity to perform daily activities. The walking speeds for the reference group in our study were comparable to those reported by Moe-Nilssen [20] for physically fit subjects with a mean age of 73 years who walked at a preferred walking speed of 3.45 km/h and a maximum speed of 5.32 km/h. Although the instructions given to the subjects were similar in both studies, our reference group walked slightly faster at both the normal and the fast walking speeds. One possible reason for this discrepancy is that our reference subjects were about 5 years younger than those in Moe-Nilssen’s study. In addition, we recruited our reference subjects from training courses and hence these subjects may have had a better individual physical fitness levels.

Male and female patients with THA showed a significant increase in walking speed at both walking speed conditions throughout the rehabilitation period, which was expected after intensive inpatient clinical rehabilitation [21]. In fact at TD3, there was no significant difference in walking speed between male patients with THA and the male reference subjects. In contrast, female patients with THA did not walk as fast as female reference subjects. One possible explanation for this discrepancy is the older age of female patients with THA compared to that of the reference subjects. Auvinet et al. [13] have previously reported decreasing walking speeds with increasing age. Further, psychological reasons may be responsible for differences at fast walking speeds in male compared with female patients with THA: male patients might be more confident and less afraid of pain or injury. In a recent study evaluating strength and motor performance in older female and male subjects (>65 years), female subjects had lower muscle strength and motor performance than male subjects even after correcting for lean muscle mass [22]. Thus, it is possible that female patients walk slower than their healthy peers to increase their perceived safety as shown in stroke patients [23, 24]. Furthermore female subjects may walk slower than their peers to avoid higher loading of the joints and muscular system. However, when asked to walk faster, female patients in our study were able to comply and reached speeds similar to those in female reference subjects (Table 2). These results are relevant for rehabilitation and we propose that the underlying impairments such as lower muscle strength and motor performance should be addressed in sex specific rehabilitation programs to facilitate faster comfortable walking speed although this requires additional study. In the context of training adaptation, a certain level of stimulus to the muscular and neuronal systems is required for improving performance and the training stimuli are greater at higher speeds.

Autocorrelation methods are good measures of symmetry or asymmetry in gait patterns [18]. In our study, only the cranio-caudal acceleration signal was used to evaluate symmetry in gait patterns. The resulting parameters P1 and symmetry index for male and female patients with THA did not reach the values of the reference group even after an intensive rehabilitation program. This result contradicts the results for walking speed. While gait symmetry parameters improved throughout the inpatient rehabilitation phase, there was still a deficit at TD3 compared to the reference group. The patient training courses comprised daily strength, mobility, flexibility and coordination training. We conclude that while this intensive training program of around 27 days improved gait symmetry in patients, this period might still be too short to achieve symmetry values of healthy, age-matched reference subjects. Results of a recent study [25] on total knee arthroplasty showed that while walking speed improved a 6-week rehabilitation program was not sufficient to achieve pre-operative values. Moreover, patients with hip osteoarthritis suffer from significant muscle strength loss and altered muscle activity [26], and it is not to be expected that these deficits would be reversed after a 4-week program.

One possible strategy for avoiding pain during gait—especially in the lower extremities—is asymmetric loading, which may expose the unaffected or less affected leg to higher ambulatory loads. When a movement is learned or has attained a high level of automation, “resetting” this pattern becomes difficult [27]. An “incorrect” adaptation may develop over a long period of time. Gait patterns specific to patients with THA presumably developed long before hip replacement with their osteoarthritic changes. Therefore, establishing a new motor program by relearning may require longer training periods and longer periods of inpatient rehabilitation than the program presented in this study. Future studies should evaluate whether a longer follow-up period after the actual treatment will completely restore symmetric gait patterns. It is important to remember, however, that a certain asymmetry can be present even in healthy subjects [8].

Specifically designed gait training programs represent one possibility to help patients achieve a symmetric gait pattern. Experienced therapists generally provide feedback on gait asymmetry in routine rehabilitation, and patients become more conscious of their movement patterns in therapy sessions. However, when unobserved, patients may return to their previous asymmetric gait, possibly because they are unaware of these subtle deviations. Using technical devices for monitoring gait could possibly minimize this deficit during unobserved periods. Systems based on inertial technology are becoming smaller, lighter and less expensive, and may hence become increasingly feasible for routine clinical use as previously suggested [12, 16].

As with most studies involving rehabilitation programs, the limitations of this study include the possibility of differences between patients by the specific exercises performed. Although we tried to standardize the program as much as possible in this inpatient rehabilitation program, there are always slight differences in the therapist-patient interaction. Six of the patients had previously received THA on their opposite hip. However, all of these patients had received their contralateral hip THA more than 1 year prior and were pain and symptom free. It is possible that asymmetry in this subsample may be a result of decreased performance on the original side or of improvements in the operated side. However, such developments would rather increase than decrease discrepancies to healthy subjects. Because these patients were pain and symptom free in the opposite side, we decided to include these patients. Moreover, in patients with unilateral THA, changes in gait asymmetry may also be caused by changes in mainly the operated or those in the contralateral limb. It is possible that assessing gait asymmetry may have influenced the motivation of our patients to perform well. Hence, the outcome in these patients may not be directly transferrable to other cohorts undergoing inpatient rehabilitation programs without additional assessments. In this study, we focused primarily on gait symmetry. However, it is well known that specific joint mechanics plays an important role in the outcome of THA, and hence should be considered in future outcome studies.

## Conclusion

Walking speed is a key parameter for determining rehabilitation progress and success and a prerequisite for regaining mobility after surgery especially for older persons. Hence, patients strive to regain a walking speed that enables them to engage in activities of daily living. Gait symmetry is another discriminative parameter of gait quality. As shown in this study, both walking speed and symmetry parameters improved during the rehabilitation period in patients following THA. However, deficits in walking speed and symmetry in patients with THA were still apparent compared to reference group suggesting a need for ongoing rehabilitation. It remains unclear if patients with THA can achieve walking speeds comparable to those in healthy subjects.

Using inertial sensor technology for assessing gait symmetry was simple and easy to evaluate, and subject compliance was high. From a methodological point of view, light-weight and small design of inertial sensor technology provides an opportunity for adapting this technology for use in gait and movement analysis and possibilities for effectively monitoring activity for controlling the outcome of the rehabilitation treatment, especially when the treatment is not performed under therapist supervision. In the current study, only coefficients from the cranio-caudal acceleration signal were analyzed, but further information can be obtained by analyzing the anterior-posterior and gyroscopic signals.
